# Dose-Effects of Aorta-Infused Clenbuterol on Spinal Cord Ischemia-Reperfusion Injury in Rabbits

**DOI:** 10.1371/journal.pone.0084095

**Published:** 2013-12-31

**Authors:** Binbin Chen, Yi Zhang, Lianhua Chen, Shiwei Huang, Shitong Li, Junyan Yao

**Affiliations:** Department of Anesthesiology, Shanghai First People's Hospital, Shanghai Jiao Tong University School of Medicine, Shanghai, China; Kaohsiung Chang Gung Memorial Hospital, Taiwan

## Abstract

**Background:**

The β_2_ adrenergic receptor (β_2_AR) plays an important role in ischemia-reperfusion (I/R) injury in various organs. Recently, a selective β_2_AR agonist clenbuterol was suggested to protect against cerebral I/R injury. This study was designed to investigate changes of β_2_ARs after spinal cord I/R injury and dose-effects of aorta-infused clenbuterol on spinal cord I/R injury in rabbits.

**Methods:**

Spinal cord ischemia was induced in New Zealand white rabbits by infrarenal abdominal aortic occlusion with a balloon catheter for 30 minutes except the sham group. During occlusion, nothing (I/R group), normal saline (NS group) or clenbuterol at different doses of 0.005, 0.01, 0.05, 0.1, 0.5, or 1 mg/kg (C_0.005_, C_0.01_, C_0.05_, C_0.1_, C_0.5_, and C_1_ groups) was infused into the occluded aortic segments. The hemodynamic data, blood glucose and serum electrolytes were measured during experimental period. Neurological function was assessed according to the modified Tarlov scales until 48 hours after reperfusion. After that, the lumbar spinal cord was harvested for β_2_AR immunohistochemistry and histopathologic evaluation in the anterior horns.

**Results:**

The β_2_AR expression in the anterior horns of the spinal cord was significantly higher in the I/R group than in the sham group. Tarlov scores and the number of viable α-motor neurons were higher in C_0.01_-C_0.5_ groups than in the NS group, C_0.005_ and C_1_ groups and were highest in the C_0.1_ group. Hypotension and hyperglycemia were found in the C_1_ group.

**Conclusion:**

β_2_ARs in the anterior horn were upregulated after spinal cord I/R injury. Aortic-infused clenbuterol (0.01–0.5 mg/kg) can attenuate spinal cord I/R injury dose-dependently during the ischemic period. The Optimal dosage was 0.1 mg/kg. Activation of β_2_AR could be a new therapeutic strategy for the treatment of spinal cord I/R injury.

## Introduction

Spinal cord ischemia–reperfusion (I/R) injury with paraplegia remains one of the most devastating complications of thoracoabdominal aortic surgery. The reported incidence of paraplegia or paraparesis ranges from 4% to 16% [1]. Although a number of strategies, including surgical techniques [2], [3], and ischemic preconditioning and postconditioning [4], reduce the risk of I/R injury, the therapeutic benefits remain uncertain.

In recent years, studies on pharmacological interventions [5] reducing spinal cord I/R injury have increased, due to greater ease of their administration. *In vivo* studies [6]–[9] showed a certain neuroprotective effect with erythropoietin, prostaglandins, and some anesthetics, but few prospective clinical trails support their efficacy in treating spinal cord I/R injury. Moreover, side effects limit their widespread application. Therefore, further investigations on drug therapy are urgently needed.

The β_2_ adrenergic receptor (β_2_AR) is proven to play an important role in cerebral ischemia [10]. Clenbuterol, a selective β_2_AR agonist traditionally used to treat asthma [11], protects against I/R injury in various organs, especially in the brain and heart [12], [13]. However, the effect of clenbuterol on spinal cord I/R injury is poorly studied. Only one recent study indicated that oral administration of clenbuterol can attenuate spinal cord I/R injury by preventing an increase in aortic blood flow after declamping [14]. In other types of spinal cord injuries, such as complete transection and contusion, clenbuterol also caused substantially enhanced recovery of locomotor function [15]–[17]. Nevertheless, the optimal dosage, time, and route of administration has not been determined.

In this study, clenbuterol was regionally infused through the aorta to get much higher concentrations in the regional spinal segment than systemic delivery [18]. The goal was to assess changes of β_2_ARs after spinal cord I/R injury, as well as to determine dose–response effects of clenbuterol on alleviating neuronal damage and improving motor function after spinal cord I/R injury.

## Materials and Methods

### 2.1 Ethic statement

All experimental procedures and protocols were approved by the Ethics Committee of Shanghai Jiaotong University (Shanghai, P.R. China) in accordance with *Guide for the Care and Use of Laboratory Animals* (U.S. National Institutes of Health publication No. 85-23, National Academy Press, Washington DC, revised 1996). Care was taken to minimize suffering for the animals. All surgical procedures were performed under general anesthesia.

### 2.2 Animals

We used 72 New Zealand white rabbits (half male and half female) aged 4–6 months weighing 2.0–3.0 kg. The animals were acclimated before the study under standard conditions for over 1 week in the Animal Research Laboratory at Shanghai First People's Hospital Affiliated to Shanghai Jiaotong University School of Medicine. All rabbits were neurologically intact before anesthesia and the surgical procedure.

### 2.3 Clenbuterol solution

Clenbuterol hydrochloride was purchased from Sigma–Aldrich (St. Louis, MO, USA). It was dissolved in normal saline and stored at 2–8°C.

### 2.4 Anesthesia and monitoring

The rabbits were fasted overnight before surgery. Ketamine (20–25 mg/kg) and atropine (0.06–0.10 mg/kg) was injected intramuscularly for basal anesthesia. A 22-G catheter was then placed into the left ear vein for fluid (normal saline containing benzylpenicillin sodium 0.48 g at a rate of 10 mL/kg/h) and drug administration. Anesthesia was induced by intravenous injection of midazolam (0.5–1.0 mg/kg). After orotracheal intubation, the animals were ventilated mechanically. Initial respiratory parameters were set as follows: ventilation mode, pressure-controlled continuous mandatory ventilation (CMV); inspiratory pressure, 10 cm H_2_O; inspiratory time, 0.8 s; respiratory rate, 30 bpm; ratio of inspiratory time to expiratory time (I: E ratio), 1∶1.4–1:1.5; and fraction of inspired oxygen (FiO_2_), 1.0. The parameters were adjusted to keep a tidal volume of 8–12 mL/kg and maintain the blood gas within the normal physiologic range. An artery in the right ear was also cannulated with a 22-G catheter to monitor hemodynamic parameters, as well as to allow for arterial blood sampling. Anesthesia was maintained with intermittent intravenous boluses of midazolam (0.4–0.5 mg/kg), fentanyl (8–10 µg/kg), and vecuronium (0.2–0.25 mg/kg). Esophageal temperature was maintained at 38°C throughout the operation, with the aid of a heating lamp.

### 2.5 Spinal cord I/R

The animals were placed in the supine position. After sterile preparation, an incision was made in the right iliac region and the right femoral artery was exposed and mobilized for 1 cm. Following a midline laparotomy, the infrarenal abdominal aorta and bilateral proximal common iliac arteries were exposed carefully through a transperitoneal approach with the abdominal contents reflected. Five minutes after systemic heparinization (1 mg/kg), a 4F Swan–Ganz balloon catheter (116F4, Edwards Lifesciences, Irvine, CA, USA), and a 20-G polycarbonate (PC) catheter was inserted in sequence through an arteriotomy in the right femoral artery and advanced about 15 cm forward into the abdominal aorta. Preliminary investigations confirmed that the balloon should be positioned just inferior to the left renal artery and the tip of the PC catheter be positioned 0.5 cm distal to the balloon.

Spinal cord ischemia was induced by abdominal aorta occlusion for 30 minutes, which was produced by inflation of the balloon with 0.5 mL normal saline. Complete aortic occlusion was confirmed by reduction in distal aortic blood pressure to less than 20 mmHg, which was measured through the PC catheter. Normal saline or different doses of clenbuterol was infused into the aorta via the PC catheter at the rate of 3 mL/kg/h for 30 minutes as soon as the aorta was occluded. At the same time, bilateral common iliac arteries were clamped to prevent drug solution leakage. The balloon was deflated and bilateral iliac arteries were declamped for the reperfusion 30 minutes later and the drug infusion was stopped in the meantime. At the end of the operation, the catheter was removed and the right femoral artery was reconstructed. The animals were monitored until they fully recovered.

After tracheal extubation, the animals were kept on a warm mat for several hours to prevent hypothermia. When completely recovered, they were singly housed in cages. Fluid supplement was given until they could ingest food and water. If the bladder was paralyzed, the rabbits were helped to urinate by pressing lower abdomen.

For preventing infection, the rabbits were given benzylpenicillin intraoperatively and postoperatively. The rearing environment was kept clean and comfortable in the whole process.

### 2.6 Experimental design

#### 2.6.1 Experiment 1

To investigate the change of β_2_ARs in the spinal cord after spinal cord I/R injury, the animals were randomly divided into 2 groups (*n* = 8 each). Sham group: The animals underwent the surgical procedure but the aorta was not occluded. I/R group: The aorta was occluded for 30 minutes with nothing infused through the aorta.

#### 2.6.2 Experiment 2

To investigate the neuroprotective effects of clenbuterol against spinal cord I/R injury and the dose–response effect, the animals were randomly divided into 7 groups (*n* = 8 each): NS, C_0.005_, C_0.01_, C_0.05_, C_0.1_, C_0.5_, and C_1_ group: Normal saline, or 0.005, 0.01, 0.05, 0.1, 0.5, or 1 mg/kg clenbuterol were administrated through the aorta during the ischemic period, respectively.

### 2.7 Physiologic parameters

The heart rate (HR), systolic blood pressure (SBP), diastolic blood pressure (DBP), and mean arterial pressure (MAP) were monitored continuously throughout the experiment. Hemodynamic data were collected at baseline (T0), at the onset of ischemia (T1), 5 minutes after ischemia (T2), 10 minutes after ischemia (T3), 15 minutes after ischemia (T4), 20 minutes after ischemia (T5), at the onset of reperfusion (T6), 5 minutes after reperfusion (T7), 10 minutes after reperfusion (T8), 15 minutes after reperfusion (T9), 20 minutes after reperfusion (T10), and 30 minutes after reperfusion (T11). Arterial blood was sampled for determination of blood glucose and serum electrolytes (potassium, sodium and calcium) at baseline (T0), 30 minutes after ischemia (T1), 30 minutes after reperfusion (T2), and 1 hour after reperfusion (T3).

### 2.8 Neurological evaluation

Immediately after the animals regained consciousness and at 6 hours, 24 hours, and 48 hours after reperfusion, hind limb motor function was assessed, by an observer who was blinded to the experimental groups, according to the modified Tarlov criteria [19]: 0 = paraplegia with no lower-extremity movement; 1 = poor lower-extremity movement, but unresistant to gravity; 2 = good lower-extremity motor function with resistance to gravity, but unable to draw legs or hop; 3 = ability to draw leg and hop, but not normally; 4 = normal lower-extremity motor function. Animals with Tarlov grades 0–1 were rated as paraplegia, while grades 0–3 were rated as neurological dysfunction.

### 2.9 Histopathology

At 48 hours after reperfusion, all animals were anesthetized and intubated as described above and placed in a left–lateral position. By means of a laminectomy, the lumbar segment (L_4-5_) of spinal cord was immediately harvested under adequate analgesia and the animals were thereafter euthanized by intravenous injection of sodium pentobarbital (200 mg/kg). The spinal cord samples were fixed in 10% formalin for 24 hours. After dehydration in graded ethanol, each segment of the spinal cord was embedded in paraffin, and cut into sections of 5-μm thickness for hematoxylin–eosin (HE) staining. Neuronal injury was evaluated with a light microscope by 2 pathologists who were blinded to the origin of the samples. One section per segment was read. Viable α-motor neurons were counted in 3 consecutive fields along a line drawn from the center of central canal to the vertex of the anterior horn (by 40× object lens), and the numbers of viable α-motor neurons in the anterior horns from each segment were aggregated, to give a total result for each animal. A viable α-motor neuron was defined according to the following criteria [20], [21]: fine basophilic granular cytoplasm with Nissl body and a soma diameter of 30–60 µm. Neurons with diffusely eosinophilic cytoplasm, loss of Nissl body and pyknotic nuclei were considered to be either dead or necrotic (red neurons).

### 2.10 Immunohistochemistry

Sections were treated with 0.3% (w/v) hydrogen peroxide in 50% methanol to eliminate endogenous peroxidase activity and then incubated overnight at 4°C with primary antibody (mouse monoclonal anti-rabbit β_2_AR antibody, 1:200, R & B, USA). After several washes in PBS, sections were incubated for 20 minutes at room temperature with secondary antibody (goat anti-mouse IgG HRP conjugate, R & B, USA). All antibodies were diluted with blocking solution. After several washes in PBS, the immunoperoxidase reaction product was visualized by diaminobenzidine (DAB) staining. The sections were then counterstained by hematoxylin, dehydrated in graded ethanol, cleared in xylene, and finally mounted. Images were viewed with Zeiss AxioCam MRc microscope and captured with AxioVision Rel 4.8 software (Carl Zeiss, Germany). Using Image-Pro Plus 6.0 software (Media Cybemetics, USA) for semi-quantitative image analysis. The protein expression of β_2_AR in the anterior horns was measured by the sum of integrated optical density (IOD_sum_).

### 2.11 Statistical analysis

Hemodynamic data, blood glucose concentration and serum electrolytes concentration at each time point were compared using 1-way analysis of variance (ANOVA) and repeated measures ANOVA. Dunnett and least-significant difference (LSD) post-hoc test was used when appropriate. The IOD_sum_ of β_2_AR expression in the anterior horns was compared using the independent-samples t-test. Tarlov scores and the number of motor neurons were compared via the Kruskal–Wallis test, followed by the Mann–Whitney U test where indicated. The incidence of paraplegia and neurological dysfunction was compared by the Chi-square test. Hemodynamic and other measurement data are presented as mean ± standard deviation (SD), the number of viable neurons is expressed as median (25th and 75th percentiles), Tarlov scores, and the incidence of paraplegia and neurological dysfunction are presented in absolute numbers. Data were analyzed using SPSS software (version 13.0, SPSS Inc, Chicago, IL, USA), and a *P* value<0.05 was considered statistically significant.

## Results

### 3.1 Experiment 1

Neurological function in the I/R group was significantly worse than in the sham group (Table 1). The Tarlov score in the I/R group was lower than that in the sham group at all intervals (*P*<0.01). Seven of 8 animals (87.5%) in the I/R group developed paraplegia at 48 hours after reperfusion, as compared with none in the sham group (*P*<0.01).

**Table 1:** Incidence of paraplegia and neurological dysfunction.

Time	Group	Incidence of paraplegia (%)	Incidence of neurological dysfunction(%)
Palinesthesia	Sham	0	0
	I/R	87.5*	100*
6 h	Sham	0	0
	I/R	75*	87.5*
24 h	Sham	0	0
	I/R	87.5*	87.5*
48 h	Sham	0	0
	I/R	87.5*	87.5*

Data are expressed as absolute numbers.

I/R = ischemia-reperfusion; 6 h = 6 hours after reperfusion; 24 h = 24 hours after reperfusion; 48 h = 48 hours after reperfusion. * *P*<0.05 vs. the sham group.

**Figure 1:**
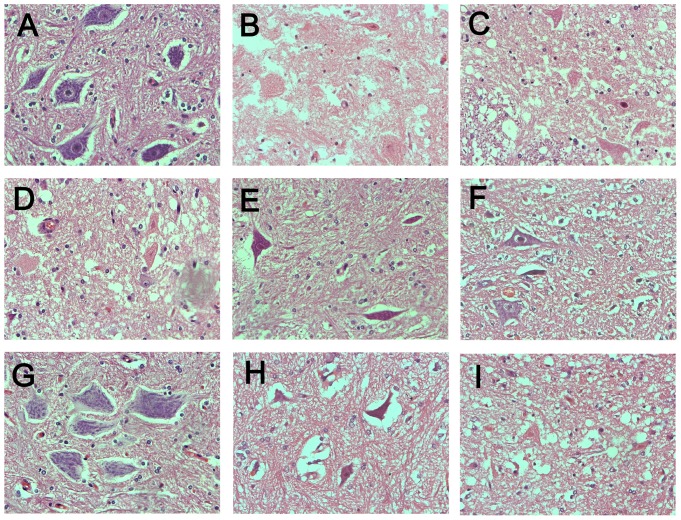
Hematoxylin and eosin staining in the anterior horn of spinal cord. (A) The sham group showed no specific histopathological change. (B) The I/R group showed marked vacuolization and significant loss of α-motor neurons. (C) The NS group showed a similar histopathological appearance to the I/R group. (E–H) Histopathological damage was attenuated in C_0.01_, C_0.05_, C_0.1_, and C_0.5_ groups. The α-motor neurons were preserved most in the C_0.1_ group. (D and I) The C_0.005_ and C_1_ groups showed no significant neuroprotection (original magnification, 40× object lens). I/R = ischemia-reperfusion; NS = normal saline; C_0.005_-C_1_ = clenbuterol 0.005–1 mg/kg.

The histopathological evaluation showed that the spinal cord was intact in the sham group, while it was severely damaged in the I/R group, as proved by vacuolization and ischemic neurons (the cell body was shrunken; pyknotic nucleus; the cytoplasm became eosinophilic, with loss of Nissl granules) (Figure 1A and 1B). The number of total viable α-motor neurons in the I/R group [0 (0–2.5)] was also less than that in the sham group [19.5 (16.75–21.5)] (*P*<0.01).

**Figure 2:**
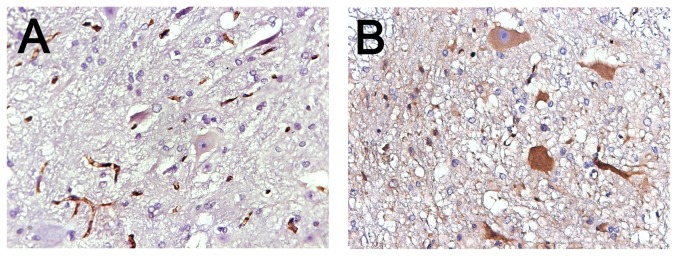
β_2_AR immunoperoxidase staining in the anterior horn of spinal cord. The positive regions were brown. (A) The sham group showed little β_2_AR expression in neurons. (B) The I/R group showed strong positive expression of β_2_AR in neurons (original magnification, 40× object lens). β_2_AR = β_2_ adrenergic receptor; I/R = ischemia-reperfusion.

The β_2_AR immunoreactivity was present at low levels in normal spinal anterior horn neurons. The I/R injury resulted in a marked increase of β_2_AR immunoreactivity. Immunostaining of β_2_AR was diffuse within α-motor neurons, and was occasionally absent in the nuclei. The IOD_sum_ of β_2_AR expression in the anterior horns of the spinal cord was significantly higher in the I/R group (22014.76±142.21) than in the sham group (141.21±50.08) (*P*<0.05) (Figure 2), indicating that spinal cord I/R injury was coincided with the upregulation of β_2_AR in the spinal cord.

### 3.2 Experiment 2

#### 3.2.1 Hemodynamics

**Figure 3:**
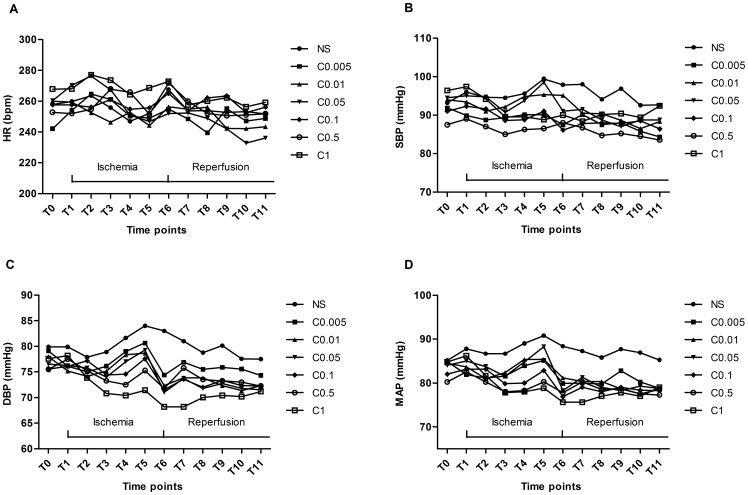
Changes of hemodynamics among different dosage groups. Clenbuterol (1 mg/kg) reduced DBP from T4 to T9, especially at T7, accompanied with the decrease of MAP. There were no significant differences in HR and SBP. NS = normal saline; C_0.005_-C_1_ = clenbuterol 0.005–1 mg/kg; HR = heart rate; SBP = systolic blood pressure; DBP = diastolic blood pressure; MAP = mean arterial pressure; T0 = baseline; T1 = the onset of ischemia; T2 = 5 minutes after ischemia; T3 = 10 minutes after ischemia; T4 = 15 minutes after ischemia; T5 = 20 minutes after ischemia; T6 = the onset of reperfusion; T7 = 5 minutes after reperfusion; T8 = 10 minutes after reperfusion; T9 = 15 minutes after reperfusion; T10 = 20 minutes after reperfusion; T11 = 30 minutes after reperfusion.

The hemodynamic data measured during the experimental period are shown in Figure 3. Clenbuterol reduced DBP and MAP, but had no significant effect on HR and SBP. The DBP was decreased in the C_1_ group from T4 to T9, especially at T7, accompanied with the decrease of MAP (*P*<0.05). Anesthetic doses and volumes of infused fluid were similar in each group.

#### 3.2.2 Blood glucose and serum electrolytes

As summarized in Table 2, clenbuterol showed a trend of dose-dependent and time-dependent increase in blood glucose concentration. In the C_0.5_ group, blood glucose levels were increased since T2, as compared with baseline (*P*<0.05). In the C_1_ group, blood glucose levels were increased at all intervals, as compared with baseline (*P*<0.05) and at T2, as compared with the NS group (*P*<0.05). Serum potassium and sodium levels were similar among all groups at corresponding time periods (*P*>0.05). Serum calcium levels were reduced in the NS group at T1 and T2 (*P*<0.05) but were not significantly changed in clenbuterol groups (*P*>0.05) (Table 3).

**Table 2:** Blood glucose concentration (mmol/L).

Group	T0	T1	T2	T3
NS	5.73±0.16	6.50±2.10	6.22±1.85	7.63±4.19
C_0.005_	5.49±0.28	7.00±3.39	7.23±2.90	7.81±2.60
C_0.01_	5.68±0.40	7.45±1.10	7.33±1.04	7.48±1.92
C_0.05_	5.35±0.07	7.20±1.51	8.03±1.11	8.53±1.50
C_0.1_	5.77±0.15	8.10±1.99	8.67±2.40	8.97±2.40
C_0.5_	5.40±0.34	7.50±1.98	8.45±1.82#	9.23±1.73#
C_1_	5.68±0.18	7.90±0.62#	8.84±1.60*#	9.22±2.03#

Data are expressed as mean ± SD.

NS = normal saline; C_0.005_-C_1_ = clenbuterol 0.005–1 mg/kg; T0 = baseline; T1 = 30 minutes after ischemia; T2 = 30 minutes after reperfusion; T3 = 1 hour after reperfusion. * *P*<0.05 vs. the NS group. # *P*<0.05 vs. before aortic occlusion.

**Table 3:** Serum electrolytes concentration (mmol/L).

	Group	T0	T1	T2	T3
Potassium	NS	3.47±0.36	3.46±0.46	3.70±0.44	3.87±0.52
	C_0.005_	3.32±0.19	3.50±0.31	3.84±1.20	3.64±0.73
	C_0.01_	3.60±0.26	3.37±0.32	3.50±0.28	3.85±0.35
	C_0.05_	3.65±0.07	3.35±0.21	3.25±0.21	3.23±0.32
	C_0.1_	3.50±0.21	3.10±0.44	3.50±0.19	3.05±0.07
	C_0.5_	3.47±0.48	3.15±0.07	3.28±0.36	3.30±0.24
	C_1_	3.54±0.29	3.48±0.23	3.80±0.52	3.80±0.69
Sodium	NS	143.67±1.58	144.00±2.60	144.11±2.32	143.22±3.63
	C_0.005_	143.11±3.06	143.00±2.40	142.44±3.43	142.33±2.12
	C_0.01_	141.33±2.31	141.67±2.08	142.33±0.58	142.33±0.58
	C_0.05_	143.00±2.83	144.00±1.00	144.33±1.53	144.00±0.00
	C_0.1_	143.50±2.12	142.50±0.71	142.00±0.00	143.50±2.12
	C_0.5_	141.20±0.84	142.33±2.34	142.67±2.58	142.33±3.01
	C_1_	143.20±2.28	144.20±2.78	144.00±1.58	142.60±2.07
Calcium	NS	1.43±0.05	1.34±0.08#	1.34±0.09#	1.38±0.11
	C_0.005_	1.44±0.09	1.37±0.08	1.41±0.13	1.39±0.13
	C_0.01_	1.37±0.12	1.28±0.14	1.33±0.21	1.34±0.15
	C_0.05_	1.42±0.17	1.35±0.14	1.37±0.17	1.41±0.21
	C_0.1_	1.35±0.04	1.35±0.09	1.31±0.16	1.42±0.03
	C_0.5_	1.36±0.10	1.32±0.12	1.39±0.14	1.47±0.16
	C_1_	1.35±0.07	1.28±0.07	1.34±0.05	1.38±0.08

Data are expressed as mean ± SD.

NS = normal saline; C_0.005_-C_1_ = clenbuterol 0.005–1 mg/kg; T0 = baseline; T1 = 30 minutes after ischemia; T2 = 30 minutes after reperfusion; T3 = 1 hour after reperfusion. * *P*<0.05 vs. the NS group. # *P*<0.05 vs. before aortic occlusion.

#### 3.2.3 Neurological outcome

**Figure 4:**
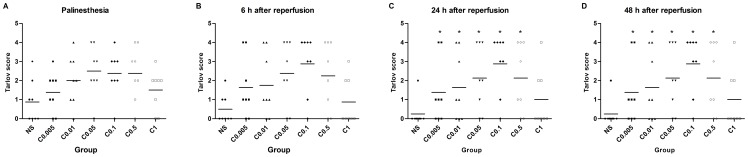
Tarlov scores at different time points after reperfusion. Tarlov scores were significantly higher in C_0.01_-C_0.5_ groups than in the NS group at 24 and 48 hours after reperfusion. The best dosage was 0.1 mg/kg. There was no significant difference among the C_0.005_, C_1_, and NS groups. Tarlov score: 0 = paraplegia with no lower-extremity movement; 1 = poor lower-extremity movement but unresistant to gravity; 2 = good lower-extremity motor function with resistance to gravity, but unable to draw legs or hop; 3 = ability to draw leg and hop but not normally; 4 = normal lower-extremity motor function. NS = normal saline; C_0.005_-C_1_ = clenbuterol 0.005–1 mg/kg; 6 h = 6 hours; 24 h = 24 hours; 48 h = 48 hours. * *P*<0.05 vs. the NS group.

All animals survived until the final neurological behavior assessment at 48 hours after reperfusion. The improvement of hind limb motor function by clenbuterol was dose-dependent. Clenbuterol showed an increasing neuroprotective effect at doses from 0.005 to 0.1 mg/kg and a decreasing neuroprotective effect at doses from 0.5 to 1 mg/kg. Tarlov scores in C_0.01_-C_0.5_ groups were significantly higher than in the NS group at 24 and 48 hours after reperfusion (*P*<0.05). There was no significant difference among the C_0.005_, C_1_ and NS groups (*P*>0.05) (Figure 4). The incidence of paraplegia and neurological dysfunction was lower in all the clenbuterol groups than the NS group at the end of observation, but was statistically significant only in the C_0.1_ group (*P*<0.05) (Table 4). The neurological outcome was best in the C_0.1_ group.

**Table 4:** Neurological and histopathological outcome at 48 hours after repercussion.

	NS	C_0.005_	C_0.01_	C_0.05_	C_0.1_	C_0.5_	C_1_
Incidence of paraplegia (%)	87.5	75	62.5	37.5	25*	50	62.5
Incidence of neurological dysfunction(%)	100	75	75	62.5	50*	62.5	87.5
Number of viable α-motor neurons	0 (0–1.75)	2 (0–6)	3.5 (3–7)*	6 (2–9.5)*	10.5 (4–12)*	5.5 (1–8.5)*	3 (0–7)

Incidence is expressed as absolute numbers. Number of viable α-motor neurons is expressed as median (25th and 75th percentiles).

NS = normal saline; C_0.005_-C_1_ = clenbuterol 0.005–1 mg/kg. * *P*<0.05 vs. the NS group.

#### 3.2.4 Histopathological outcome

**Figure 5:**
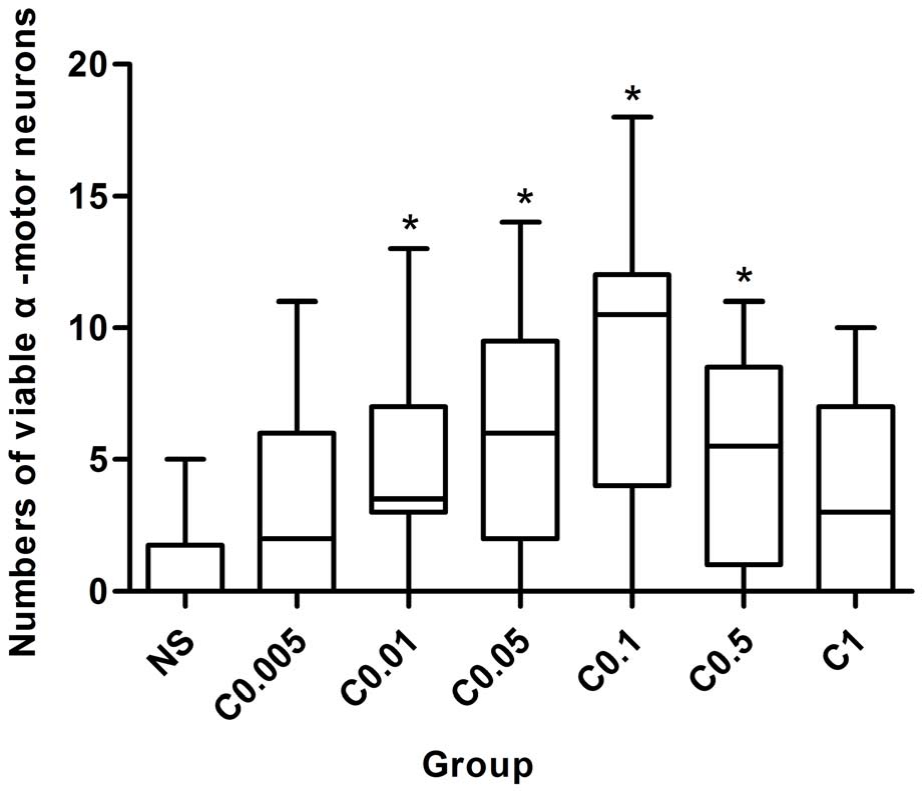
Numbers of viable α-motor neurons in the anterior horn of spinal cord. The numbers of viable α-motor neurons were greater in C_0.01_-C_0.5_ groups than in the NS group. The best dosage was 0.1 mg/kg. There was no significant difference among the C_0.005_, C_1_, and NS groups. NS = normal saline; C_0.005_-C_1_ = clenbuterol 0.005–1 mg/kg. * *P*<0.05 vs. the NS group.

The trend of histopathological outcome was consistent with neurological scores. As shown in Figure 2C–2I, treatment with clenbuterol attenuated the histopathological damage of spinal cord in a dose-dependent manner as described in 3.2.3. The numbers of viable α-motor neurons were greater in C_0.01_-C_0.5_ groups than in the NS group (*P*<0.05) (Table 4 and Figure 5). The neuronal loss was least in the C_0.1_ group. No significant differences were found among the C_0.005_, C_1_ and NS groups (*P*>0.05).

## Discussion

The present study demonstrated that β_2_ARs in the anterior horn were upregulated after spinal cord I/R injury. Administration of clenbuterol 0.01–0.5 mg/kg through the aorta during the ischemic period protected the spinal cord against I/R injury in a dose-dependent manner, and the protective effect is most significant at the dose of 0.1 mg/kg.

A rabbit model of spinal cord I/R injury is widely used because of the segmental blood supply of its lumbosacral spinal cord [22]. The lumbosacral spinal cord receives blood exclusively from the infrarenal abdominal aorta and thus is susceptible to ischemic injury when this segment is occluded [23]. In this study, spinal cord I/R injury was produced by abdominal aorta occlusion for 30 minutes by means of inflation of the balloon instead of conventional aortic clamping. This modification made the surgical procedure easier as well as attenuated the hemorrhage and lymphatic fistula resulted from abdominal aorta mobilization [18]. Clenbuterol was infused into the occluded aorta segment. The biggest advantage of regional drug administration is that a much higher concentration of clenbuterol can be delivered directly to the ischemic segment of the spinal cord via the lumbar arteries. This method enhances the protective effect on the spinal cord and minimizes the adverse effects on other organs in comparison with systemic administration [24], [25].

The β_2_AR is proven to play a key role in I/R injury. In models of myocardial ischemia, increased β_2_AR expression is beneficial against I/R injury, according to studies on transgenic animals overexpressing β_2_ARs or with knockout for β_2_ARs [26]–[29]. In this study, we defined the distribution of β_2_ARs in the spinal cord. By using immunohistochemistry, we showed that β_2_ARs were expressed mainly in the anterior horn with low level before ischemia, which complemented previous findings in rats [30]. After I/R injury, a marked increase of β_2_AR immunoreactivity was detected in α-motor neurons of the anterior horn, indicating that β_2_AR expression is significantly upregulated after spinal cord I/R injury. The β_2_ARs were mainly distributed in the cell membrane and cytoplasm of α-motor neurons. The distribution of β_2_ARs was also detected in the in the nuclei but was occasionally absent. This phenomenon indicated that the upregulated expression of β_2_ARs is likely to act as a response to I/R tolerance.

Whether inhibiting or stimulating β_2_AR contributes to neuroprotection is controversial [10]. It is reported that brain injury is reduced and neurological outcome improved after transient focal cerebral ischemia in mice lacking the β_2_AR, or in wild-type mice pretreated with a selective β_2_AR antagonist [31], [32]. On the other hand, a larger number of earlier studies indicated the neurologic benefits of β_2_AR agonists [12], [33]–[39]. Clenbuterol, a lipophilic long-acting β_2_AR agonist that can readily cross the blood-brain barrier, reduced ischemic brain damage and neuronal death in models of both permanent and transient cerebral ischemia. *In vitro*, clenbuterol protected the cultured cerebral cortex and hippocampus neurons against glutamate-induced excitotoxic damage [33]. *In vivo*, treatment with clenbuterol resulted in an improved neurological outcome as well as a decreased infarct volume and the number of damaged neurons [12], [34]. The neuroprotective effect of clenbuterol against cerebral ischemia injury was dose-dependent [35], [37]. These conflicting results with β_2_AR may result from differences in injury types, dosage, time, and route of administration.

Unfortunately, the studies on clenbuterol against I/R injury have insufficient reported results in the spinal cord. Only one recent study by Lee et al. [14] indicated that clenbuterol protects against paraplegia after thoracoabdominal aortic surgery in rabbits when treated orally (9 mg given in drinking water) 24 hours prior to ischemia. As for other types of spinal cord injuries, such as complete transection and contusion in rats, clenbuterol has been shown to spare spinal cord tissue and enhance locomotor recovery by posttreatment of oral administration for a long period at the dose of 1.6 mg/kg/day or a single intraperitoneal injection at a dose of 2 mg/kg [15]–[17]. In this study, β_2_AR expression was upregulated after I/R injury, suggesting that increased β_2_AR expression may contribute to neuroprotection. Upon these bases, we concluded that stimulation of β_2_AR can protect against spinal cord I/R injury and the results supported our hypothesis.

Dosages of clenbuterol was investigated at 6 graded levels from 0.005 to 1 mg/kg in this study. The findings revealed that clenbuterol showed a neuroprotective effect in spinal cord I/R injury in a dose-dependent manner. Treatment with clenbuterol attenuated hind-limb motor dysfunction and histopathologic changes in spinal cord I/R injury for 48 hours. The therapeutic benefits were increasing at doses from 0.005 to 0.1 mg/kg and decreasing at doses from 0.5 to 1 mg/kg. Both the neurological and histopathological outcome were best at the dose of 0.1 mg/kg. The incidence of paraplegia at the end of observation was 25% at the optimal dose. It was similar to that (38%) found by Lee et al. [14]. The somewhat lower incidence of paraplegia may be due to the modifications in dosage, time, and route of administration.

In our study, clenbuterol was consecutively administrated as soon as the aorta occlusion was implemented so that the regional drug delivering can cover the whole ischemic period and provide direct protection to the ischemic spinal cord segments. We also recognized that once the aorta blood flow restoration beginning, the clenbuterol remainder would release to the circulation as a bolus, which may give additional protection against the reperfusion injury. The therapeutic window following injury is markedly longer in the spinal cord compared to the brain [16]. Clenbuterol afforded neuroprotection only when administered several hours prior to or immediately after injury in permanent and transient cerebral ischemia [12], [33], [35]–[38], while the therapeutic window of clenbuterol was prolonged to 48 hours postinjury in spinal cord contusive injury. These results correspond in time to the acute phase of traumatic spinal cord injury that is considered to be a period lasting 1–2 days postinjury in which inflammation, devascularization, and cell death are prominent [16]. The reason may be related to the more extensive and longer time course of inflammation in the injured spinal cord relative to the brain [40]. Our findings complemented the condition of I/R injury in the spinal cord. In our former study on a rabbit model of spinal cord I/R injury, we found out that 80% of the animals developed paraplegia after 30 minutes of ischemia and hindlimb dysfunction developed within 48 hours after reperfusion with no remarkable change thereafter (published in Chinese), which was consistent with others' previous studies [41]–[44]. In our preliminary study, animals treated with clenbuterol were observed for a maximum for 3 weeks, the neurological outcome did not differ from 48 hours after reperfusion to the endpoint. Therefore, the period of postoperative observation was 48 hours in this study. Clenbuterol is proven to have a long-lasting neuroprotection up to 7 days after transient cerebral ischemia [12] and 6 weeks after spinal cord contusion [16].

However, clenbuterol treatment also had some adverse effects. Reduction of the DBP and MAP and increased blood glucose concentration were main side effects found in the animals that were treated with 1 mg/kg clenbuterol. The HR, SBP, and serum electrolytes (potassium, sodium and calcium) concentration were not significantly affected by clenbuterol. It has been shown that a reduction of the mean arterial blood pressure to 60 mmHg increased the ischemic damage after focal cerebral ischemia [45]. In addition, increased blood glucose concentration has been suggested to accelerate tissue acidosis during ischemia and aggravate the neuronal damage [46]–[48]. These could explain why treatment with 1 mg/kg clenbuterol caused a smaller neuroprotective effect than the lower dosage. This result was consistent with the previous studies [12], [35]. It was also reported that clenbuterol administration could result in hypokalemia [49], but this phenomenon was not found in this study. Severe adverse effects such as heart toxicity were not observed because they only occur after repeated application of high doses of clenbuterol over days [50].

The mechanism underlying the neuroprotective effect of clenbuterol on spinal cord I/R injury is not fully understood. Lee et al. [14] found out that in a rabbit model of spinal cord I/R injury, post-aortic clamping paraplegia was associated with hyperemia. The neuroprotective effect of clenbuterol was related to prevention of an increase in aortic blood flow after declamping. Reperfusion initiated with low-pressure perfusion exerts neuroprotective effects on the spinal cord against I/R injury [51]. In a rat model of spinal contusive injury, Zeman et al. [15], [16] pointed out that the enhanced locomotor function due to clenbuterol was associated with sparing of spinal cord tissue at the contusion center, particularly of myelinated white matter. Clenbuterol may oppose neurodegeneration by improving redox status at the spinal cord contusion site as indicated by a glutathione-dependent increase in glutathione reductase activity during the acute phase and reduction in protein carbonyl accumulation during the following subacute phase. Glutathione was involved in a number of factors with neuroprotective properties such as nerve growth factor and Bcl-2. Moreover, clenbuterol can promote axonal regrowth after complete spinal cord transection in a long term observation [17]. Stimulation and inhibition of β_2_AR may lead to different signal pathways.

There are several limitations in this study. First, all drugs were administrated during the ischemic period. Groups in which clenbuterol is administrated before ischemia or after reperfusion will be included in further study to determine the time–response effect of clenbuterol. Second, it is unclear whether the upregulation of β_2_ARs after spinal cord I/R injury results in the initiation of neuronal damage or neuronal repair. Further studies will be designed to elucidate the underlying mechanism of the protective effect by clenbuterol.

In conclusion, this study demonstrated that β_2_ARs in the anterior horn were upregulated after spinal cord I/R injury. Clenbuterol 0.01–0.5 mg/kg infused through the aorta can attenuate spinal cord I/R injury dose-dependently during the ischemic period. The neuroprotective effect is most significant at the dose of 0.1 mg/kg. Activation of β_2_AR could become new therapeutic strategy for the treatment of spinal cord I/R injury.
